# The Dangers of Exploratory Puncture of the Chest

**Published:** 1905-10-28

**Authors:** 


### THE DANGERS OF EXPLORATORY PUNCTURE OF THE CHEST.

Dr. Joseph Wilks has related in the "British Journal of Children's Diseases" a fatal outcome to the exploratory puncture of a child's chest on account of a suspected empyema. The facts are as follows: A child of three years had undergone an attack of measles six months before, since which he had never really recovered his health. On examination he was slightly febrile, fairly nourished, and showed some clubbing of the fingers. The heart was slightly displaced to the left, and the whole of the left side of the chest gave, upon auscultation and percussion, signs compatible with the presence of an empyema. The chest was therefore punctured in the eighth space below the angle of the scapula, and a few drops of curdy pus were withdrawn. Almost immediately a stream of dark blood issued by the needle: the child gave a cough and expectorated a small quantity of dark blood upon the pillow. There was no attempt at respiratory effort; the pupils dilated and the sphincters became relaxed, and, in spite of all restoratives, the child died. Post-mortem it was found that the whole of the left lung was in a condition of tuberculous consolidation; the stomach contained blood which had been swallowed, but it was calculated that not more than six ounces had been lost in all. The heart was fatty, and the opinion of Dr. Wilks is that the child died of heart failure due to his debilitated condition.

The journal from which this case is quoted takes the opportunity of founding upon this text a not untimely warning against the indiscriminate use of the needle for the purposes of diagnosis. It may be granted that exploratory puncture of the chest is in general a safe procedure. We have ourselves performed a large number without calamity, but there are on record sufficient accidents in the course of the operation to justify us in the regard of puncture as a late resource. The question of risk hardly arises in cases where the presence of fluid is certain and where the function of the puncture is nothing more than to determine whether the fluid be pus or not. In such cases the only danger to be apprehended is the wounding of an intercostal vessel, which reasonable caution should enable us to avoid. But in cases where the presence of a collection of fluid is in doubt, where, that is to say, the diagnosis rests between a small loculated empyema and a fibrosing or chronically inflamed lung, exploration may well lead to disaster from lesions of pulmonary vessels by the entering needle, or, as Dr. Russell and Dr. Brodie have pointed out, from reflex cardiac inhibition brought about by stimulation of afferent fibres of the vagus nerve distributed in the lung. It becomes therefore necessary to weigh the relative benefits accruing to a successful attempt at the discovery of such an empyema, and to the expectant treatment of such an empyema supposing it to exist. Now it may be admitted at once that incision and drainage of such an abscess is the best treatment, and leads to the most rapid recovery. On the other hand, post-mortem experience makes it quite certain that small empyemata are spontaneously cured, by inspissation and gradual absorption, and that without leaving any very gross legacy of injury to the neighbouring lung. The evidence therefore points to the wisdom of "giving nature a chance" where the presence of fluid is a matter of reasonable doubt. Each case must of course be treated on its merits, and with due regard to the upward or downward tendency which its progress exhibits; but the editorial in question is certainly right in asserting that the modern tendency to regard needling of the chest as an almost routine procedure, and as almost a substitute for careful physical examination, is highly to be reprehended.

None the less there will always remain cases which, like the one detailed above, are not cases of empyema and which yet imitate it so closely that exploration becomes a duty. In such circumstances we can do no more than hope for the best.

